# Barriers to treatment and care for depression among the youth in Uganda: The role of mental health literacy

**DOI:** 10.3389/fpubh.2023.1054918

**Published:** 2023-03-02

**Authors:** Kennedy Amone-P'Olak, Adrian Ivan Kakinda, Henry Kibedi, Bernard Omech

**Affiliations:** ^1^Department of Psychology, Kyambogo University, Kampala, Uganda; ^2^Department of Public Health, Lira University, Lira, Uganda

**Keywords:** depression, barriers, mental health literacy, youth, Uganda

## Abstract

**Background:**

Depression represents a significant mental health problem (MHP) in low- and middle-income countries (LMICs), especially among early adults. Nevertheless, most early adults with depression do not seek treatment. Failure to recognize depression and knowledge about mental health literacy (MHL) may contribute to poor help-seeking behavior. This study assessed MHL, access and barriers to mental health care for depression among early adults in Uganda.

**Methods:**

Data were collected from students in two of the largest universities in Uganda. Participants completed questionnaires on depression, MHL, sources of barriers and opportunities for MH service. Regression analyses and parametric tests were used to assess depression, access, barriers and opportunities to promote MH.

**Results:**

About 12 per cent (*n* = 56) of the respondents were at the level of moderately severe to severe depression. Mental health literacy (MHL) scores were generally low ranging from “Ability to recognize mental disorders” (*mean* = 19.32, *SD* 3.22, *range* 18–32), and “Knowledge of risk factors” (*mean* = 4.39, *SD* 1.17, *range* 2–8), and “Knowledge of available information” (*mean* = 9.59, *SD* 2.53, *range* 5–20). Respondents reported barriers such as “stigma/discrimination” (65.53%), “lack of knowledge of where to receive help” (65.15%), “lack of trust in health workers” (62.56%), and “distant health facilities” (19.70%) that impede access to treatment and care. MHL significantly predicted depression (based on a continuous scale) (*β* = 0.63, 95% confidence interval [CI]: [0.56, 0.70]) with the regression model yielding a significant fit [*R*^2^ = 0.40, *F* (2, 460) = 189.84, *p* < 0.001].

**Conclusions:**

MHL is low among university students amidst several barriers such as stigma, fear, and lack of trust. To attenuate the negative effects of MHPs on wellbeing and lower the increased risk of psychopathology into adulthood, it is critical to prioritize MHL, address barriers to treatment and care, and develop the requisite infrastructure to tackle depression among early adults.

## Key messages

### What is already known on this topic

- The high prevalence of depression among early adults is widely recognized.
- Mental health literacy is low among early adults.

### What this study adds

- To attenuate the harmful effects of MHPs on depression and lower the increased risk of psychopathology into adulthood, it is critical to prioritize mental health literacy and address barriers to treatment and care.

### How this study might affect research, practice or policy

- Prevention and intervention programs for early adults suffering from depression may benefit from addressing mental health literacy and tackling barriers to treatment and care to reduce the toxic effects of depression among early adults.
- Tailored psychoeducation and self-help programs may potentially reduce depression among early adults.

## Introduction

According to the World Health Organization (WHO), 8% (≈ 586 million) of the total world population suffers from mental illness (1). Most of the people suffering from mental health problems, particularly early adults, are in low and middle-income countries (LMICs), where 85% of the world's population live and 80% of people with mental illnesses are found (2, 3). In Uganda, several factors have been implicated in the development and sustenance of mental disorders such as depression. These factors include, *inter alia*, poverty (4), diseases (5), unemployment (6), and war (7). Depression also represents a risk factor for other mood disorders and substance abuse (8). Moreover, the burdens of depression are increased since most people with depression never seek treatment or usually do so late. Consequently, knowledge about mental health problems (MHPs) such as depression, mental health literacy, and barriers to mental health care is crucial to developing interventions to address MHPs.

In Africa, Uganda is among the countries with high rates of MHPs such as depression and anxiety disorders (9). The prevalence of depression is estimated at 4.6% and anxiety at 2.9% (1, 10–12), which sometimes are associated with suicidal behaviors (13). Therefore, MHPs are a major public health concern in Uganda yet the country spends only about 10% of its gross domestic product on healthcare and a minuscule 1% on mental healthcare (10). Most of this funding is spent at the national mental health referral facility at Butabika Hospital in the capital city, leaving the majority of those suffering from mental illness in the rural areas without any care or service (10) except the mental health services provided by the non-governmental organization (NGOs).

Most of the population in LMICs, especially early adults, experience several barriers to mental health care including low MHL and poor access to mental health services. MHL is defined as knowledge and beliefs related to mental disorders that help in diagnosing, managing, and preventing mental health problems and is recognized as an important predictor of favorable mental health outcomes (14, 15). Thus, improved MHL is essential for early access, diagnosis, and interventions to alleviate psychological distress, particularly among adolescents and early adults (15–17). Even though MHL has been widely studied in Europe and North America, it remains under-studied in many LMICs including Uganda, particularly among early adults (18). In Uganda, we found only one study by Okello et al. (18) on mental health literacy among early adults, which highlighted poor knowledge and attitudes of early adults toward mental illness.

Low MHL, negative attitudes, and barriers to accessing mental health care limit access to treatment and care for early adults with MHPs (19). Failure to recognize depression as an illness requiring treatment and knowledge that the treatment can be beneficial may lead to poor help-seeking behaviors. Yet, improved MHL has been associated with prevention, increased access to mental health services, early diagnosis, early treatment and better treatment outcomes (1). A study to increase public awareness about depression was associated with positive attitude change that improves help-seeking (20).

This study is rooted in the theory of MHL developed by O'Connor and Casey (21). This theory is based on six pillars: (1) the ability to recognize specific disorders or different types of psychological distress, (2), knowledge of risk factors and causes, (3) knowledge of self-treatment, (4) knowledge of professional help available, (5) knowledge of where to seek information, and (6) attitudes that promote recognition or appropriate help-seeking behavior. The theory was used as a framework within which to explain the unique needs and circumstances of early adults regarding their mental health problems.

With a population of about 44 million people, it is estimated that 14 million people in Uganda have mental illness representing about 32% of the total population (22) up from a previous estimate of about 24% a decade ago (23). Moreover, primary healthcare in Uganda is weak and under-resourced in terms of funding, human resources, and infrastructure, thus being unable to meet the mental health needs of the people (22). Furthermore, Uganda has only 53 psychiatrists countrywide; many of whom are placed in towns and cities leaving the rural areas where the majority of the population lives without mental healthcare workers (22, 23).

For this reason, this study aimed to evaluate the MHL for depression in a sample of early adults enrolled in universities in Uganda. Specifically, the current study will: (1) assess MHL, (2) measure the level of depression, (3) assess barriers to care for depression, and (4) quantify the extent to which MHL predicts depression among early adults. Previous studies indicate that depression is more common among women than men (24) and that more women access and use mental health services than men (25), thus the current study will also examine gender differences regarding MHL and depression.

## Method

### Design and sample

We conducted a quantitative study using cross-sectional design to assess the levels of MHL and depression, barriers to care and treatment, and the associations between MHL and levels of depression among early adults following various courses in tertiary education in 2022. In this study, a regression statistical analysis using G^*^Power 3.1.9.2 software was used to compute the sample size (26). Based on an effect size of 0.8, a significance level of α = 0.05, and a statistical power of 1–*β* = 0.8, the power analysis indicated a sample size of 450 students enrolled in different study programmes. Originally, 492 students were requested to participate in the study, 17 of whom refused to take part, seven (7) were underage, and (6) inadequately responded to the items in the questionnaires. Accordingly, data from 462 participants, representing a response rate of 94 per cent, of those originally invited were included for analyses.

### Procedure and ethical considerations

Lecturers and faculty administrators granted permission to research assistants to distribute questionnaires during class hours in the lecture rooms and theaters. The research assistants explained the purpose of the study to the students and informed them about the anonymity, confidentiality, and voluntary nature of their participation in the study before the questionnaires were distributed to the students. Next, the questionnaires were handed to students who agreed to take part in the study. In addition, the participants were requested not to indicate any identifying mark on the questionnaire. The questionnaire took about 10 min to fill out. Ethics approval for the current study was granted by Makerere University Ethics Committee in 2022. All participants provided written informed consent before the questionnaire was handed to them.

### Measures

In this study, a self-report questionnaire comprising the following three sections: (1) socio-demographic questions (age, gender, year of study, parental educational attainment, and (2) Mental Health Literacy Scale (MHLS) developed by O'Connor and Casey (21), and (3) the nine-item Patient Health Questionnaire (PHQ-9) depression scale (27, 28) were used. Barriers to care for depression were assessed by 11 items included in Table 3.

### Patient health questionnaire-9 (PHQ-9)

The 9-item Patient Health Questionnaire (PHQ-9) (27, 28) is a widely used depression screening instrument used in non-clinical settings. The PHQ-9 is based on the 9 *Diagnostic and Statistical Manual of Mental Disorders, 4th Edition* (DSM-IV) criteria of major depression measured on a 4-point Likert-type scale from “0” (not at all) to “3” (nearly every day) assessing depressive symptomatology over the previous 2 weeks. The total score on the PHQ-19 ranges from 0 to 27, with high scores meaning high depression. The scores are interpreted as follows: no depression (0–4), mild (5–9), moderate (10–14), moderately severe (15–19), or severe (20–27). A cut-off score of 10 is suggested as indicating a possible diagnosis of depressive disorder. The PHQ-9 has previously been used in Uganda with excellent psychometric properties (29).

### The mental health literacy scale (MHLS)

The Mental Health Literacy Scale (MHLS) consists of 35 questions categorized into six dimensions: (1) ability to recognize disorders, (2), knowledge of risk factors and causes, (3) knowledge of self-treatment, (4) knowledge of available professional help, (5) knowledge of where to seek information, and (6) attitudes that promote recognition or appropriate help-seeking behavior. MHLS was initially developed by O'Connor and Casey (21).

#### Ability to recognize disorders

This attribute consists of eight questions that were measured using a 4-point Likert scale (very unlikely, unlikely, likely, very likely). This attribute refers to “the ability to correctly identify features of a disorder, a specific disorder, or category of disorders”.

#### Knowledge of risk factors and causes

This attribute was measured with two questions and using a 4-point Likert scale (very unlikely, unlikely, likely, very likely). This attribute refers to “knowledge of environmental, social, familial or biological factors that increase the risk of developing a mental illness”.

#### Knowledge of self-treatment

This attribute consists of two questions that were measured using a 4-point Likert scale (very unhelpful, unhelpful, helpful, very helpful). This attribute refers to “knowledge of typical treatments recommended by mental health professionals and activities that an individual can conduct”.

#### Knowledge of professional help available

This attribute was measured with three questions and using a 4-point Likert scale (very unlikely, unlikely, likely, very likely). This attribute refers to “knowledge of mental health professionals and the services they provide”.

#### Knowledge of where to seek information

This attribute consists of four questions that were measured using a 5- option Likert scale (strongly disagree, disagree, neither agree nor disagree, agree, strongly agree). This attribute refers to “knowledge of where to access information and capacity to do so”.

#### Attitudes that promote recognition or appropriate help-seeking behavior

This attribute consists of sixteen questions and was measured using a 5-option Likert scale (strongly disagree, disagree, neither agree nor disagree, agree, strongly agree) or (definitely willing, probably willing, neither willing nor unwilling, probably unwilling, definitely unwilling). This attribute refers to “attitudes that impact on the recognition of disorders and willingness to engage in help-seeking behavior”.

The 35 MHLS items are grouped as follows: the ability to recognize disorders (8 items), knowledge of where to seek information (4 items), knowledge of risk factors and causes (2 items), knowledge of self-treatment (2 items), knowledge of professional help available (3 items) and attitudes that promote recognition or appropriate help-seeking behavior (16 items). The MHLS has yielded very good psychometric properties concerning internal consistency, content and structural validity (30) and internal and test-retest reliability (21). The internal consistency of the MHL questionnaire as evaluated by Cronbach's alpha was good and acceptable at (Cronbach's alpha = 0.873) (21). While there are items related to the relevant attributes, they are often considered together.

We performed principal component analyses (PCAs) using the direct oblimin rotation method for the MHL scale based on scores of the total sample (*N* = 462) to confirm the factor structure of the instrument. The eigenvalues in the scree plot (31) and the eigenvalue rule (32) were employed to decide on the number of factors in the MHL scale. The construct validity was computed based on the 35 items that constitute the MHL scale. A Kaiser–Meyer–Olkin (KMO) value obtained was 0.71 (recommended value is 0.60). The Bartlett's Sphericity test outcome was χ^2^ (43) = 1,239, *p* < 0.001, indicating sufficient correlation structure to carry out the factor analyses employing the PCA extraction technique. Subsequently, a PCA with a direct oblimin rotation technique with a cut-off of 0.30 and using the Kaiser's (32) criterion of eigenvalues yielded a six-factor solution as the best fit for the data, accounting for 67.1% of the variance. Seven- to eight-factor solutions explained only 8 and 4% of the variance, respectively.

### Data analysis

Descriptive statistics (mean, *SD*, and range) were run to assess the levels of MHL, depression and barriers to care for depression. Domains of MHL and total MHL were standardized to a mean of 0 and standard deviation (SD) of 1 (z scores) before performing all the analyses. Univariate regression models were fitted to assess the extent to which each of the domains of MHL and total MHL scores predicted depression based on a continuous scale. Finally, a multivariate regression model was fitted to assess whether the MHL domains that significantly predicted depression in univariable models uniquely and independently predicted depression based on a continuous scale. The severity of depressive symptoms was used as the dependent variable in a one-way analysis of variance (ANOVA) with the different domains of MHL as the independent variable. Tukey HSD test for multiple comparisons was used to examine significant differences between levels of severity of depression. To examine the possible modifying effect of age and sex, we assessed the interaction between the total MHL X age and the total MHL X sex on depression as a continuous variable. All analyses were conducted using Mplus version 8 (33) with a *p*-value of 0.05 to indicate the level of significance.

## Results

### Socio-demographic characteristics of study participants

The overall socio-demographic characteristics of the study population are presented in Table 1 based on data obtained from 462 participants (male 268, 58%). The average age of participants was 22.39 (*SD* = 2.13, range: 18–25) years with male students (*M* = 23.83, *SD* = 1.78) significantly older than their female peers (*M* = 21.25, *SD* = 1.96; *t* = 4.53, *p* < 0.01).

**Table 1:** Socio-demographic characteristics, mental health literacy and depression among study participants.

	**Total**		**Male**		**Female**		
	**Mean/no**	**(SD/%)**	**Mean/no**	**(SD/%)**	**Mean/no**	**(SD/%)**	***t*** **-test**
Participants	*N* = 462	100%	*N* = 268	58%	*N* = 194	42%	
Age	22.39	SD = 2.13	23.83	SD 1.78	21.25	SD 1.96	2.52, *p* < 0.05
	**Mean (SD)**	**Range**	**Mean (SD)**	**Range**	**Mean (SD)**	**Range**	
Mental health literacy subscales	62.87 (7.42)	(29–130)	58.51 (6.83)	(29–130)	67.23 (6.31)	(29–130)	5.29, *p* < 0.001
Ability to recognize disorders (8 items)	19.32 (3.22)	(8–32)	18.01 (3.52)	(8–32)	20.63 (2.52)	(8–32)	
Knowledge of risk factors (2 items)	4.39 (1.17)	(2–8)	3.84 (1.07)	(2–8)	4.94 (1.23)	(2–8)	
Knowledge of self–treatment (2 items)	4.05 (0.81)	(2–8)	3.17 (0.84)	(2–8)	4.93 (0.98)	(2–8)	
Knowledge of available help (3 items)	5.13 (1.15)	(3–12)	4.37 (1.15)	(3–12)	5.89 (1.78)	(3–12)	
Knowledge of available information (4 items)	9.59 (2.53)	(5–20)	8.26 (2.71)	(5–20)	10.92 (2.73)	(5–20)	
Help-seeking attitudes (16 items)	33.58 (4.28)	(16–80)	31.57 (5.30)	(16–80)	35.59 (5.11)	(16–80)	
Depression (PHQ-9 scores)	25.05	SD = 14.46	17.69	SD 13.08	28.09	SD 13.93	4.72, *p* < 0.001
	**Frequency**	**%**	**Frequency**	**%**	**Frequency**	**%**	
Minimal or no depression (0–4)	*N* = 241	52.2	*N* = 156	58.2	*N* = 85	43.8	
Mild depression (5–9)	*N* = 98	21.2	*N* = 55	20.5	*N* = 43	22.2	
Moderate depression (10–14)	*N* = 67	14.5	*N* = 35	13.1	*N* = 32	16.5	
Moderately severe depression (15–19)	*N* = 41	8.9	*N* = 17	6.3	*N* = 24	12.4	
Severe depression (20–27)	*N* = 15	3.2	*N* = 5	1.9	*N* = 10	5.1	

N/n, number; SD, standard deviation.

### Levels of MHL and prevalence of depression among study participants

Similarly, the different levels of MHL are presented in Table 1. Compared to previous studies, the levels of MHL were generally low in all domains of the MHL questionnaire (Table 1). Similarly, female participants scored higher than their male peers on all domains of MHL (Table 1) demonstrating that female students in tertiary education were generally more literate than their male peers regarding mental health. On the contrary, female students scored higher than their male counterparts on depression. About 12 per cent (*n* = 56) of the respondents were at the level of moderately severe to severe depression.

### Univariable regression analyses of domains of MHL and total MHL and depression

Table 2 presents the results of the univariable regression models of domains of MHL and total MHL on depression. Overall, all the domains of MHL significantly predicted depression in separate analyses (based on a continuous scale) in univariable regression analyses (Table 2). Comparative Fit Indices (CFI) ranged from 0.96 to 0.98, and root mean square error of approximation (RMSEA) ranged from 0.04 to 0.06 for the models for the separate models. Often, CFI values (0.96) and RMSEA values of 0.06 indicate excellent model fit (34, 35).

**Table 2:** Univariable regression model of the unique influence of different domains of mental health literacy and total mental health literacy on depression based on a continuous scale.

**Mental health literacy**	***β* (SE)**	***p* < value**	**CFI**	**RMSEA**
Age	0.02 (0.04)	ns	0.95	0.06
Sex	0.21 (0.04)	0.01	0.95	0.06
Knowledge of risk factors	0.41 (0.04)	0.01	0.96	0.05
Ability to recognize disorders	0.34 (0.04)	0.01	0.95	0.06
Help-seeking attitudes	0.33 (0.04)	0.01	0.95	0.06
Knowledge of available information	0.33 (0.04)	0.01	0.96	0.05
Knowledge of self-treatment	0.28 (0.04)	0.01	0.96	0.06
Knowledge of available help	0.25 (0.04)	0.01	0.95	0.05
Total MHL	0.63 (0.04)	0.01	0.97	0.05

*β*, standardized beta; SE, standard errors; CFI, comparative fit indices; RMSEA, root mean squared error of approximation; ns, not significant; MHL, mental health literacy. All significant associations are in bold.

### Multivariate regression analyses of domains of MHL and depression

Table 3 presents the results of the multivariable regression models aimed at assessing the extent to which the different domains of MHL independently and uniquely predict depression. The outcome of this analysis indicated that “knowledge of risk factors”, “ability to recognize disorders”, “help-seeking attitudes”, and “knowledge of available information” independently predicted depression. Likewise, the CFI was 0.96 and RMSEA was 0.05 for the multivariable regression model (Table 3).

**Table 3:** Multivariable regression model of the unique influence of different domains of mental health literacy on depression based on a continuous scale.

**Mental health literacy**	***β* (SE)**	***p* < value**	**CFI**	**RMSEA**
Sex	0.15 (0.06)	0.05	0.95	0.05
Knowledge of risk factors	0.23 (0.04)	0.01		
Ability to recognize disorders	0.18 (0.05)	0.05		
Help-seeking attitudes	0.17 (0.06)	0.05		
Knowledge of available information	0.16 (0.06)	0.05		

*β*, standardized beta; SE, standard errors; CFI comparative fit indices; RMSEA, root mean squared error of approximation; ns, not significant.

### Moderating effects of age and sex on depression

Sex and age did not significantly modify the effect of MHL on depression. After entering the interaction terms of age with total MHL and sex and total MHL the interaction terms did not confer any additional significant influence on depression among the early adults. The results of the interaction analyses are not presented but were as follows: age X total MHL: *β* = −0.08, 95% CI (−0.16, 0.01) and sex X total MHL: *b* = 0.06, 95% CI (−0.03, 0.15).

### Barriers to seeking help for depression

Participants who scored higher (scores 15–27) and were in the range of moderately severe to severe depression as measured by PHQ-9 reported significantly more barriers to care than those who scored lower (i.e., scores between 0 and 14) (Table 3). The majority reported a lack of trust in help providers', “stigma”, lack of knowledge about where to get help, and “fear” (Table 4).

**Table 4:** Perceived barriers to seeking help for depression stratified by scores 0–14 and 15–27 on PHQ-9.

**S/No**	**Barriers to seeking help for depression**	**PHQ-9 scores (0–14) (*n* = 406)**	**PHQ-9 scores (15–27) (*n* = 56)**	**χ^2^ (*1*, *N* = 462) *p-value***
1	Fear of stigma/discrimination from the community	262/406 (65.53%)	42/56 (75.00%)	**χ**^2^ = 48.11, *p* < 0.05
2	Do not know where to receive help from	261/406 (65.15%)	39/56 (69.64%)	**χ**^2^ = 58.43 *p* < 0.05
3	Fear of break-up	257/406 (63.30%)	37/56 (66.07%)	**χ**^2^ = 97.28, *p* < 0.05
4	Lack of trust in help providers	254/406 (62.56%)	43/56 (76.79%)	**χ**^2^ = 6.47, *p* < 0.05
5	Busy (studying, business, etc.)	253/406 (62.32%)	36/56 (64.29%)	**χ**^2^ = 61.30, *p* < 0.05
6	Counseling does not help much	200/406 (49.26%)	35/56 (62.50%)	**χ**^2^ = 23.05, *p* < 0.05
7	Do not know where to go	194/406 (47.78%)	34/56 (60.71%)	**χ**^2^ = 35.42, *p* < 0.05
8	Financial difficulties	169/406 (41.63%)	32/56 (57.14%)	**χ**^2^ = 57.33, *p* < 0.05
9	Fear of problems worsening	159/406 (39.16%)	29/56 (51.79%)	**χ**^2^ = 36.12, *p* < 0.05
10	Distant health facility (hospital)	80/406 (19.70%)	27/56 (48.21%)	**χ**^2^ = 18.45, *p* < 0.05
11	Traditional healers	63/406 (15.52%)	13/56 (23.21%)	**χ**^2^ = 9.41, *p* < 0.05

PHQ-9–9-item patients health questionnaire.

## Discussion

From the onset, the current study aimed to assess MHL, how it was associated with depression and the barriers to accessing services to mitigate depressive symptoms among students pursuing higher education in Uganda. The current study showed low levels of mental health literacy, high levels of depressive symptomatology, and many barriers to accessing services that may improve knowledge of why early adults with depression do not access mental health services. The fact that well-educated early adults in institutions of higher learning could have such low levels of MHL serves as a yardstick for later studies on MHL for depression and other MHPs for folks in the community. Previous studies showed that participants with low MHL were 3 times more likely to have depression (36). Furthermore, there were gender differences regarding MHL and depression. The gender differences highlight the need to factor gender in intervention designs.

The results of this study suggest that, indeed, low MHL predicts adverse mental health problems such as depression. All domains of MHL significantly predicted depression (see Table 2). Consequently, improved mental health literacy skills may inform interventions to reduce depression that is common among early adults in institutions of higher learning where MHPs appear to be on the increase (7, 37).

### Agreement with previous studies

The results of this study agree with previous studies that MHL predicts poor mental health outcomes such as depression (36). Regarding the prevalence of depression, our findings are in tandem with previous findings with similar rates of depression among students pursuing higher education in tertiary institutions (7, 37). If we took the cut-off points of a score of 10 on the PHQ-9 questionnaire, those with moderate to severe depression were 26.6 per cent (*n* = 123) and those with moderately severe and severe depression were 12.1% (*n* = 56). On the other hand, MHL was very low for all the dimensions of the MHL questionnaire (38).

On the predictors of depression by different dimensions of MHL, we could not point to specific dimensions as the main predictors because many of them were significantly correlated in preliminary analyses indicating that they were possibly working in tandem to predict depression. There may be several mechanisms through which depression and MHL may be related. For instance, lack of knowledge about MHPs (e.g., depression), stigma and low access to services have been associated with poor help-seeking behaviors (39).

### Limitations and strengths of the study findings

The results of this study may be limited in many ways. First, the cross-sectional design may inherently include biases arising from self-report measures. Second, the homogenous sample of university students (e.g., in terms of age, education, or income) may not be representative of early adults out of school, thus, it may be difficult to extrapolate the results to other samples of early adults outside school. Third, the source of information for both MHL and depression is the same. Consequently, this might have led to inflated coefficients as a result of biases arising from the same source variance. Last, other factors such as sources of depression, background characteristics, and anxieties related to the COVID-19 pandemic were not assessed or adjusted for in the analyses. These factors might have inflated depressive symptoms. Nevertheless, the current study adds to the scanty literature on mental health literacy in low- and middle-income countries, particularly among early adults. Finally, we employed the robust multivariate rigorous structural equation modeling technique known to reduce measurement errors, increasing the credibility of our results. Furthermore, the Mplus software is recognized to be robust enough to limit the effects of type II errors (34, 35). The fit indices in the Mplus software such as RMSEA (0.06) and CFI (0.95) yielded acceptable limits for this study (34, 35).

### Implications of the findings

Knowledge about the low levels of MHL and depression and that low levels of MHL predict depression are crucial in planning interventions to educate early adults about common mental disorders and access mental health services. With the increase in depression and other common mental disorders, more research and interventions are required to understand the sources of common mental disorders and to educate and empower early adults to reduce the toxic and long-term effects of mental illness. Knowledge of common mental disorders is critical in creating awareness and accessing mental health services. Nonetheless, it is imperative to be aware that improving knowledge alone may not be adequate to increase the uptake of mental health services. Stigma and other barriers to treatment and care need to be tackled too. The results of this study should herald further longitudinal studies with bigger sample sizes to delineate the role of MHL in the awareness and access to mental health services among students in institutions of higher learning. Mainly, research should emphasize MHL in overcoming the barriers to treatment and care such as lack of trust, stigma, and knowledge of where to receive help, among others. Programs on interventions to reduce depression should target students with various forms of life challenges such as academic stress, relationship difficulties, and financial stress, among others. The early adult population at risk of serious depression needs to be treated, so higher education institutions should set up counseling clinics and psychosocial support services.

## Conclusion

The lack of policy frameworks that address MHPs, stigma, fear, and limited access to mental health professionals and services are some of the factors contributing to the low level of MH literacy among university students. Opportunities exist for simple, less costly and easy-to-follow stress management techniques offered by non-professionals. Developing MH infrastructure to address MHPs and interventions to address low MHL and poor attitudes to increase service use is required. To attenuate the negative effects of MHPs on depression and lower the increased risk of psychopathology into adulthood, it is critical to prioritize MHL, address barriers to treatment and care, and develop the requisite infrastructure to tackle depression among early adults.

## Data availability statement

The raw data supporting the conclusions of this article will be made available by the authors, without undue reservation.

## Ethics statement

The studies involving human participants were reviewed and approved by Makerere University Human Research Ethics Committee. The patients/participants provided their written informed consent to participate in this study.

## Author contributions

KA-P'O was responsible for this study, conceived, and designed the study. KA-P'O, BO, AIK, and HK participated in preparing the manuscript, read, and approved the final manuscript. All authors contributed to the article and approved the submitted version.
